# Ultra-stable speckle-based optical fiber sensing demonstrated on an uncrewed aerial vehicle platform

**DOI:** 10.1038/s44172-026-00603-w

**Published:** 2026-02-05

**Authors:** Przemyslaw Falak, Toby King-Cline, Akos Maradi, Timothy Lee, Bruno Moog, Pawel Maniewski, Robert Entwistle, Martynas Beresna, Christopher Holmes

**Affiliations:** 1 Optoelectronics Research Centre, University of Southampton, Southampton, Hampshire UK; 2 School of Engineering, University of Southampton, Southampton, Hampshire UK; 3 Department of Applied Physics, KTH Royal Institute of Technology, Stockholm, Sweden

**Keywords:** Optical sensors, Imaging and sensing, Mechanical engineering

## Abstract

Speckle-pattern interrogation offers a route to high-resolution spectral sensing, but its uptake has been constrained by poor temporal stability under real-world conditions. Here, we introduce an ultra-stable speckle-based architecture that overcomes these limitations and enables real-time structural health monitoring of uncrewed aerial vehicles. Unlike conventional approaches that rely on large-scale, free-space passive speckle decorrelation, our system utilizes an ultra-compact speckle pattern via laser-written scattering centers in a high aspect ratio flat fiber, encapsulated within a 3D-printed polylactide housing. This architecture suppresses environmental drift and enables robust, high-fidelity interrogation of fiber Bragg gratings in dynamic aerospace conditions. The system demonstrated exceptional stability under sustained mechanical excitation, maintaining measurement integrity at ±7 G sinusoidal acceleration along the axial direction. Furthermore, in-flight validation across uncrewed aerial vehicle flight tests confirmed real-time strain interrogation in the −100–400 µε range with a standard deviation in measurement of 1.63 µε. These results mark the demonstration of stable, real-time speckle-based interrogation in flight, establishing a path toward broader deployment of specklemeters in harsh environments.

## Abstract (web summary)

Przemyslaw Falak and colleagues report optical speckle–based fiber sensing demonstrated outside the lab on a drone under vibration and environmental disturbance. The study shows specklemeter technology can support structural monitoring of real-world aerospace systems

## Introduction

The growing use of uncrewed aerial vehicles (UAVs) in delivery, surveillance, and defense has created a pressing need for automated, in-flight structural inspection systems^1–3^. This is particularly important for enabling safe and reliable Beyond Visual Line of Sight (BVLOS) operations. With limited ground crew availability, automation enables UAVs to operate more frequently and independently, improving overall fleet operational efficiency and utilization. Continuous in-flight monitoring supports data-driven maintenance strategies to ensure flightworthiness, reduce downtime and operational costs. Among the available technologies, optical fiber sensing stands out as a highly promising solution due to compact size, light weight, as well as optical signal immunity to electromagnetic interference (EMI). EMI immunity is especially valuable in small aircraft, where dense electronic systems may generate noise that compromises signal fidelity.

Fiber Bragg gratings (FBGs) are cost-effective, compact, optical sensors based on wavelength selective reflectors, where the central Bragg wavelength is sensitive to changes in temperature or strain^4^. Their advantages include low cost, multiplexing capability, and compatibility with fiber-optic systems. FBG based sensing systems are widely employed for real-time structural health monitoring in civil engineering^4,5^, aeronautics^6^, and submersibles^7^. Nonetheless, interrogation of FBGs on small and lightweight UAVs is a challenge, as existing solutions cannot offer satisfactory SWaP-C (space, weight, power, and cost).

Traditionally, FBG interrogation techniques rely on either wavelength scanning or dispersion-based methods, which convert spectral information into the temporal or spatial domain. Dispersion-based approaches, such as Czerny-Turner spectrometers, are often bulky, while newer integrated photonic devices remain expensive in small volumes due to fabrication complexity. Wavelength scanning techniques, which use tunable lasers or filters, typically increase system cost, power consumption and physical footprint. Furthermore, these interrogation methods require a clean, high-contrast FBG spectrum, as peak tracking often relies on Gaussian fitting or center-of-mass algorithms. Consequently, spectral distortions from fabrication imperfections or environmental influences can compromise peak detection accuracy and increase measurement error.

One emerging approach to satisfy stringent SWaP-C challenges is specklemeter-based interrogation, which offloads hardware complexity into virtual space, leveraging machine learning (ML) and artificial intelligence (AI) algorithms to improve SWaP-C metrics. Specklemeters utilize reconstructive spectrometry approaches, that exploit generated wavelength-dependent speckle patterns for spectral measurements by spectral-to-speckle mapping (i.e., matching a given spectral input with a unique output speckle pattern image). Since the establishment of the spectral reconstruction paradigm, numerous approaches have been developed to realize such a mapping, as any medium capable of inducing scattering could potentially be integrated into the system. Notable examples include randomly distributed rough alumina powder^8^, integrating sphere^9^, multi-mode optical fibers (MMF)^10^ and 2D photonic chips^11^. For instance, in the case of MMF-based approach, spectral interrogation system demonstrated up to attometer spectral resolution^12^. Such high spectral resolution offered by multiple scattering or mode-coupling process and simple lensless design makes this solution particularly attractive for space-confined applications. Moreover, unlike the conventional spectral-based measurement techniques, scattering-based reconstructive interrogation bypasses the need for explicit spectral decoding.

However, widespread deployment of these systems has been limited by a fundamental trade-off between resolution, stability, and SWaP-C constraints^13^. A critical bottleneck lies in the temporal instability of scattering media used. Reconstructive spectral measurement relies on prior data training and assumes physical invariance to maintain a stable, repeatable mapping between input wavelength and output speckle pattern. Perturbations, such as bending, twisting, vibration or temperature fluctuations reduce measurement fidelity.

To assess the temporal stability of specklemeter signals, it is necessary to characterize the evolving spatial distribution of refractive index fluctuations. These fluctuations arise from dynamic environmental perturbation, such as temperature changes and strain induced by vibration or applied loads. Consequently, the time varying speckle intensity *I(t)* is governed by the accumulated optical phase (Eq. 1).

1 $$I(t)\propto {\int }_{\!\!\!0}^{L}n(\varPhi (r,t)){dl}$$

Where *r* denotes the spatial trajectory of the optical path, Φ*(r,t)* is a scalar field that modulates the local refractive index due to environmental perturbation. In specklemeter architectures employing extended optical pathlengths, such as those based on MMF, light propagates through a spatially distributed medium, integrating refractive index variations across uncorrelated regions. When the strain or temperature field, Φ, exhibits non-negligible spatial gradients, a broad sampling increases susceptibility to temporal instability in the speckle intensity *I(t)* and the resulting speckle pattern variance. Folded optical architectures, such as those based on scattering media, reduce the stochastic sensitivity of the system to environmental perturbations by confining extended path lengths within compact scattering volumes.

Maintaining speckle pattern invariance is especially challenging, even in controlled environments, due to stochastic sensitivity of the system to subtle, spatial distribution perturbations. This issue is often addressed through advanced spectral reconstruction algorithms that leverage ML and AI, particularly neural network-based image recognition techniques^14–16^. Nonetheless, previous demonstrations have frequently relied on tightly controlled conditions and repeated calibration procedures to ensure consistent spectral measurements.

Recently, our team demonstrated that scattering media can be laser-written into 3D free-space coupled silica plates^17^, and non-circular optical fiber platforms^18^. The latter design simultaneously exploits two wavelength-sensitive processes: mode coupling and optical scattering, to reduce the total volume of the system and thus effectively reduce the accumulation of uncorrelated environmental perturbations and thereby reducing temporal speckle pattern variation. Under stable, controlled laboratory conditions (22–25 ^°^C), temporal stability of up to 7 days, wavelength resolution of 0.05 nm^17^ and strain resolution of 2 µε for FBG interrogation^18^ were achieved.

Here, we present a fully integrated Speckle-based Tracking and Stabilized Interrogation System (STASIS), an ultra-stable specklemeter, constructed using high-aspect-ratio flat fiber (HARFF) and incorporating a laser-written scattering matrix. HARFFs represent a new class of optical fibers, defined by a quadrilateral cross-section with a width to thickness aspect ratio (AR) of 10:1 or greater^19^. The HARFF used in this work had an AR of 25:1 with 40 µm thickness and less than 1 µm peak-to-valley surface variations. Such ultra-thin, ultra-flat cross-section enhances optical confinement and supports stable, low-noise speckle generation. Moreover, the HARFF was embedded within a custom 3D-printed stabilization, which acts to further mechanically isolate the fiber-detector interface and preserves system alignment, contributing to long-term stability even under exposure to mechanical shock and vibration.

The system’s stability was validated on a mechanical shaker, maintaining reliable performance under axial accelerations up to ±7 G across vibration frequencies from 5 to 60 Hz. The proof-of-concept STASIS architecture was demonstrated in a real-world application i.e., in-flight monitoring of a 2 m wingspan UAV. Multiple UAV flights confirmed real-time strain interrogation in the −100–400 µε. The system’s stability enabled the use of a simple *dissimilarity* metric for reconstruction, avoiding the complexity of multivariate methods while also serving as a direct indicator of the interrogator’s intrinsic stability. The results demonstrated in this work establish a clear pathway for cost-effective, speckle-based sensing technologies for deployment in harsh environments and across a broad range of industrial applications.

## Results

### Test flights and stability validation

**Fig. 1:**
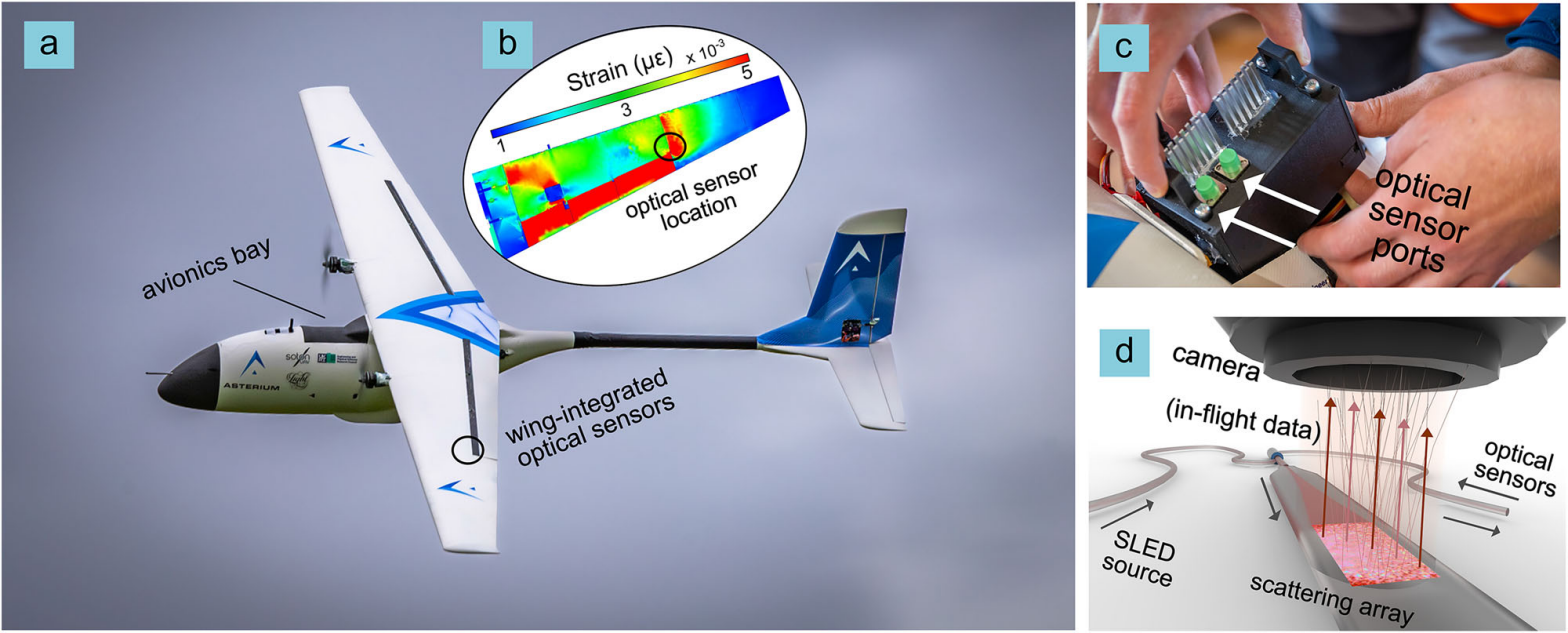
Overview of speckle-based grating interrogator for drone flight test. **a** Photograph of uncrewed aerial vehicle used for test flights. Optical sensors were integrated in high strain areas of wings based on **b** finite element analysis modeling. **c** Photograph of interrogator head unit placed within avionics bay of uncrewed aerial vehicle, and **d** sketch of working principle. SLED superluminescent diode.

To demonstrate the potential for in-flight structural heath monitoring, STASIS was used to track strain at key locations on the UAV’s wings. Fiber Bragg gratings were embedded at locations of highest predicted in-plane strain, identified through finite element analysis, Fig. 1a, b (see “Methods”). The STASIS unit was housed in the avionics bay shown in Fig. 1c and interfaced with the UAV’s autopilot, enabling real-time data streaming via telemetry link.

In contrast to conventional speckle-based systems that often rely on free-space optics and discrete optical components, this compact monolithic platform employs a continuous, physically connected optical path, eliminating alignment-sensitive free-space elements and thereby improving robustness and integrability under dynamic flight conditions. HARFF is fusion-spliced directly to standard circular fiber, forming a fully fiberized platform that combines multimode propagation with internally defined scattering features in a compact geometry (Fig. 1d). This reduced system volume, acts to limit environmentally induced dimensional perturbations, as expressed by Eq. 1. HARFF geometry not only improves optical confinement but also enables seamless mechanical integration within a 3D printed stabilization housing, further reinforcing structural robustness during dynamic operation (“Methods”).

### STASIS performance evaluation under vibration and temperature

**Fig. 2:**
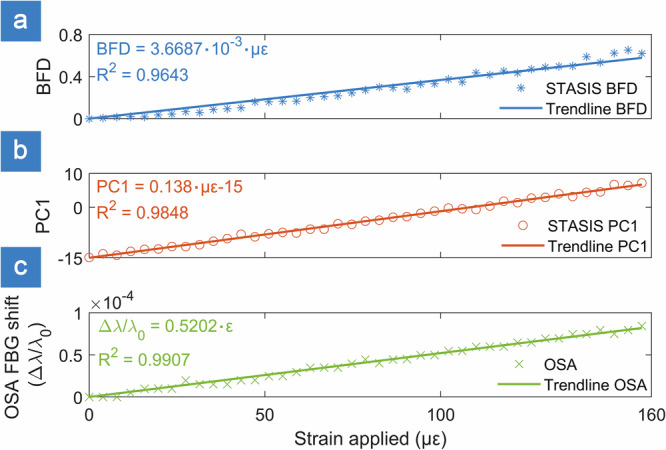
Comparison of fiber Bragg grating response to applied strain when analyzed using different techniques. **a** Speckle-based tracking and stabilized interrogation system (STASIS) baseline frame dissimilarity (BFD) method; **b** STASIS first principal component (PC1) projection method and **c** direct fiber Bragg grating (FBG) wavelength shift measured by an optical spectrum analyzer (OSA), used as industry-wide standard.

Speckle transformation induced by strain on the FBG were assessed using two alternative approaches (see “Methods”). In one case, the speckle pattern variation is evaluated with Baseline Frame Dissimilarity (BFD) metric, which is based on scalar product between two vectors. In the second case, the variation is evaluated via first principal component PC1 obtained using Principal Component Analysis (PCA). Responsivity to the strain for both methods was evaluated by comparing against spectral data captured by an optical spectrum analyzer (OSA) (Fig. 2). Both speckle metrics indicate a linear relation to the applied strain.

**Fig. 3:**
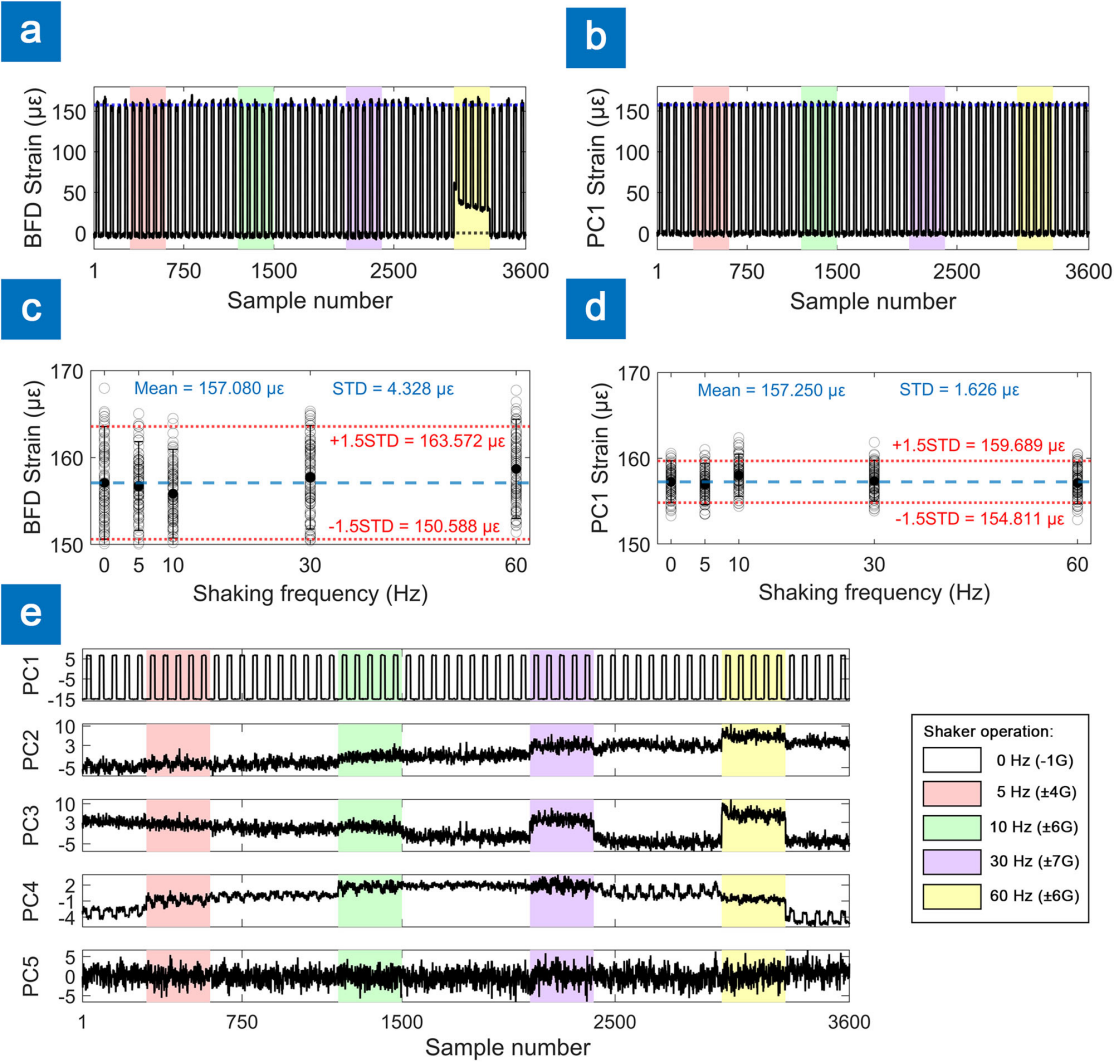
Interrogator head vibration experiments. **a** Strain derived from baseline frame dissimilarity (BFD) and **b** from the first principal component (PC1) evaluated during shaking. Colored backgrounds mark shaker operation at different frequencies (white: stationary; pink: 5 Hz; green: 10 Hz; purple 30 Hz; yellow: 60 Hz). **c** Statistical analysis of BFD strain and **d** PC1 strain. Dashed blue line indicates mean strain; dotted red line indicates ±1.5 standard deviation (STD) strain range. **e** First 5 principal components (PCs 1–5).

To simulate representative operational flight conditions for on-board systems and assess system stability, the STASIS head was subjected to controlled external perturbation, in the form of sinusoidal mechanical vibrations with frequencies ranging from 5 to 60 Hz with peak acceleration from ±4 to ±7 G (Fig. 3). The frequency range was chosen to be above and below STASIS sampling frequency (10 Hz). The signal traces acquired during the vibration tests demonstrate the system’s ability to maintain performance under dynamic loading (Fig. 3).

A set of strain measurements were performed while subjecting STASIS head to controlled vibrations. The PC1-derived strain exhibits a resting standard deviation of STD = 1.63 µε. As illustrated in Fig. 3(d), induced vibrations at 5, 10, 30, and 60 Hz all fall within a ± 1.5 STD of this resting baseline.

At higher excitation frequencies (notably 60 Hz), the BFD exhibits clear baseline lift due to the external vibrations, confirming that this scalar measure is sensitive to dynamic perturbation. In contrast, the PCA-based representation remains largely unaffected. Vibration-induced changes map onto higher-order components orthogonal to the wavelength-sensitive PC1 direction. This separation is evident in Fig. 3a–d, where the PC1 trajectory is preserved across all tested vibration levels. These results demonstrate that although the raw speckle similarity can be perturbed by vibration, the PCA decomposition effectively decouples these effects and provides a more stable basis for tracking wavelength shifts.

**Fig. 4:**
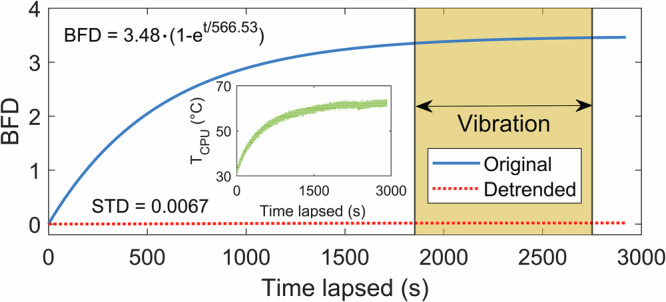
Speckle-based tracking and stabilized interrogation system performance during warm up from cold start. Baseline frame dissimilarity (BFD) of speckle patterns (blue solid line) and detrended data (red dotted line) against time. STD denotes standard deviation of detrended BFD trace. Orange shaded region indicates 30 Hz ±4 G mechanical shaking. Insert shows on-board processor temperature *T*_CPU_ inside interrogator unit over the same period.

Environmental tests revealed that the BFD exhibits a deterministic exponential saturation following a cold start, with a time constant *τ*_*speckle*_ = 566.53 s (Fig. 4), and reaching a steady state value (BFD ~ 3.5) within 30 min. Notably, this trend closely mirrored the thermal response of the Raspberry Pi CPU, which increased from 30 to 65 °C in 30 min at a rate of ^*τ*^_*temp*_ = 565.88 s (Fig. 4). This correlation indicates that the observed dynamics of the BFD is driven by an increasing temperature of internal electronic components (CPU and CMOS sensor) and can therefore be effectively detrended as a predictable interrogation unit warm-up artifact. The effectiveness of this operation and mathematical model applied can be seen in Fig. 4. When vibrations were introduced following the thermal warm-up phase, the BFD output remained consistent with that observation under static conditions, indicating that the STASIS platform’s performance was unaffected by mechanical excitation and that the speckle pattern remained stable, even under this representative environmental extreme. All subsequent BFD data is temperature-compensated using the integrated thermistor signal as a reference for temperature within the interrogation unit.

### Flight testing specklemeter subsystem

Following successful stability validation of STASIS, under controlled mechanical vibration, a series of UAV test flights were conducted to evaluate the in-flight performance of the specklemeter subsystem. Each test flight consisted of five phases: (A) runway positioning, (B) take off, (C) steady-state orbiting, (D) aerobatic maneuvers and (E) landing. An illustrative flight path is shown in Fig. 3a, covering a total distance of 14.34 km. In this work, we employed and compared two distinct strategies for in-flight monitoring: (i) Principal Component Analysis (PCA) projection, and (ii) BFD algorithm (see “Methods”). For each flight, speckle patterns were captured at the frame rate of 10 fps. Prior to take-off, the UAV was left idle for approximately 30–50 s, during which speckle patterns were collected and used to compute a baseline average (DC-level) that was subsequently subtracted from in-flight speckle frames to normalize measurements.

**Fig. 5:**
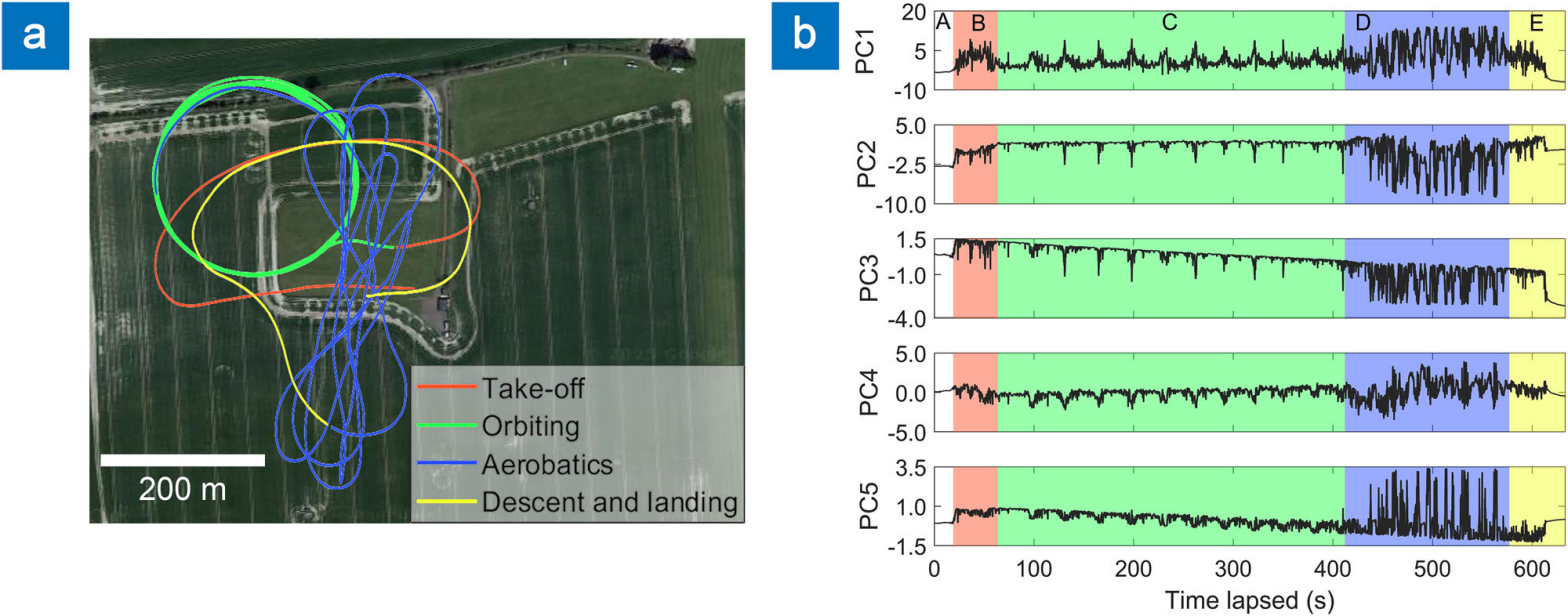
Flight test and principal component analysis results. **a** Flight map with overlaid colored paths representing flight phases: (A, white)-idle aircraft at the runway, (B, red)-take-off and altitude climbing, (C, green)-autonomous circular orbiting, (D, blue)-aerobatics and (E, yellow)-landing and touchdown); **b** projection of first five principal components (PC1–PC5) during flight. Colored sections and letterings refer to the distinct flight phases. Map background by Google Maps (imagery by Maxar Technologies, 2025), used in compliance with Google Maps Platform and Geo Guidelines (https://about.google/brand-resource-center/products-and-services/geo-guidelines/).

During each flight, both the vertical acceleration readings obtained with the autopilot unit (The Cube Orange, 50 Hz sampling rate) and the strain measured via the FBGs (at 10 Hz sampling rate) were collected. For PCA projection, during the lift-off (30 s), captured speckles were used to compute PC space, capitalizing on the rapid and gradual increase in wing strain to define the basis vectors. The top five contributing principal components, derived from the test flight data, are shown in Fig. 5b, for two spectrally multiplexed FBGs collocated on a single wing (see Methods). Temporal evolution of these components clearly reflects the different flight phases. The first principal component (PC1), which accounted for the dominant variance in the speckle data, exhibited values ranging from −9 to 15 (note, PC projection is unitless in the abstract space). The remaining components followed similar temporal trends, but with lower absolute magnitudes. Notably, several components, including PC3 and PC5 exhibited baseline drift, which correlates with STASIS system performance in controlled environment and is attributed to warming of the Raspberry Pi CPU. However, given that their amplitudes were at least an order of magnitude smaller and their variance contributions were approximately six times lower than PC1, their overall influence was weaker by a factor of 60, contributing a negligible amount to the total captured signal variation. Consequently, PC1 was selected for strain monitoring. Its values were linearly scaled and calibrated using the pre-recorded strain calibration data (see “Methods”).

**Fig. 6:**
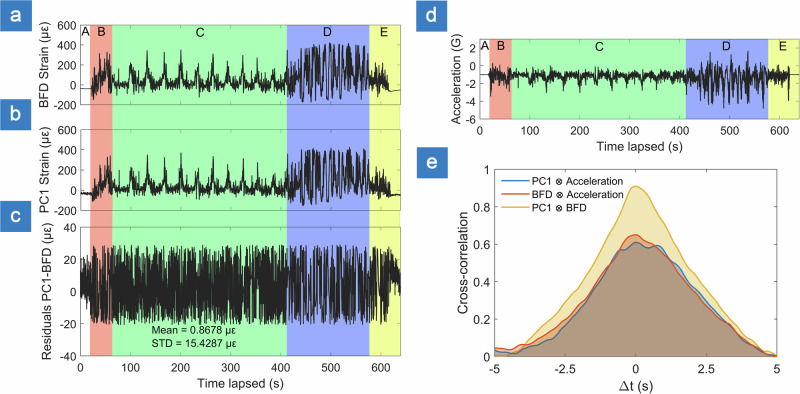
Analysis of reconstructed strain data from flight test. **a** Baseline frame dissimilarity (BFD) derived and **b** first principal component (PC1) derived strain signals recorded during uncrewed aerial vehicle flight. **c** Residuals between two reconstructed strain signals, STD denotes standard deviation of residuals. **d** In-flight vertical acceleration captured by on-board accelerometer. Colored regions represent flight phases: (A, white)-idle aircraft at the runway, (B, red)-take-off and altitude climbing, (C, green)-autonomous circular orbiting, (D, blue)-aerobatics and (E, yellow)-landing and touchdown). **e** Cross-correlation of PC1 and BFD strain signals with accelerometer data and with one another.

Both the vertical acceleration (Fig. 6d) and strain readings (Fig. 6a, b) clearly reflect distinctive features of the five flight phases described earlier. The measured acceleration was proportional to the strain experienced by the drone, with the proportionality factor of −0.004 and an *R*^2^ value of 0.514. During the initial idling phase, stable baseline values of approximately −1 G (gravitational acceleration) and −50 µε strain were observed, indicating minimal external loading on the wings aside for the crosswind effects (~10 m/s). In the subsequent climbing phase, vertical acceleration ranged from 0 to −2.5 G, while strain increased from −50 to 300 µε, corresponding to dynamic loading imposed during ascent. Next, during the orbiting, there were periodic increase-decrease sequences of the acceleration and strain were recorded. Here, evident periodicity (−1 to −3 G, 0 to 300 µε) was linked to the strong unidirectional wind (~10 m/s). For instance, when the aircraft faced the wind direction, the acceleration increased, while when its flight path was aligned with the wind direction, the acceleration decreased. The fourth flight phase (aerobatics) had characteristic variation with high peak-to-valley values for the acceleration and strain. Here, the acceleration varied from +1.8 G to −4G, and strain increased from 0 to 400 µε in 10 s. The final phase (landing and the touchdown) readings exhibited acceleration from −0.5 to −2 G, and strain from 0 to 200 µε with the peak of −3.8 G at the moment of touchdown and −50 µε. The remaining flat line corresponded to the idle acceleration/strain after the UAV had landed.

To enable a direct comparison between PCA-based reconstruction and BFD method, the same in-flight datasets were evaluated using both approaches. Strain values presented in Fig. 6a were obtained using BFD scaling (data referenced to the initial speckle pattern), while Fig. 6b shows the evolution of strain calculated using PC1 (see “Methods”). The quantitative difference between two reconstructed signals is evaluated by calculating residual and cross-correlation (Fig. 6c, e). Residual signals show high frequency noise like variation on the level of ±20 με. Both BFD and PC1 have similar cross-correlation values with acceleration, which is 0.6. The main difference arises from high frequency noise present in acceleration signal. The comparison of PC1 with BFD reveals a high level of correlation reaching 0.92. The consistent agreement between the BFD and PC1-derived strains across all flight phases is an indication that the speckle pattern response remains stable under operational conditions, and that even a simple matrix operation can reliably track the underlying strain.

## Discussion

The performance of the STASIS platform under controlled vibration, thermal drift and flight testing provides clear assessment of speckle stability. Across all tests, the fully fiberized optical system demonstrates ability to decouple influence of environmental perturbations to a degree that is not typical for speckle interrogation systems.

The vibration experiments show that mechanical excitation couples differently into the two reconstruction metrics, because they probe distinct aspects of the speckle field. BFD responds directly to any change in the speckle pattern and therefore acts as a diagnostic of the fundamental stability of the optical field. At low excitation frequencies (≤30 Hz) its variation is minimal, consistent with mechanical stability of the system, whereas at 60 Hz an elevated baseline reflects induced perturbation of the underlying speckle distribution. PCA, however, successfully isolates the wavelength-dependent component of the speckle evolution to a single principal component. Vibration-induced changes map into orthogonal, higher-order components, leaving PC1 trajectory unchanged across all tested conditions. Thus, while BFD reveals when the speckle field itself is being perturbed, PCA separates this modal distortion from wavelength drift, evidenced by consistent FBG tracking over all induced vibration conditions.

Thermal behavior follows a deterministic and therefore removable trend. The saturated warm-up of the interrogation unit is reflected in the BFD metric, occurring on the same timescale as the 30–65 °C rise in the CPU and CMOS sensor, indicating that the dominant effect is internal heating rather than optical instability. Once temperature is compensated using the thermistor-derived model, the residual BFD signal matches the behavior seen under fully stabilized conditions. Importantly, PCA remains largely unaffected during this period. The thermally induced speckle drift maps into higher-order components, leaving the wavelength-sensitive PC1 direction unchanged.

The thermal effect on the FBGs and its influence to the strain readings was not investigated as the focus of the demonstration was monitoring of the dynamic strain variation. Temperature referencing can be implemented by adding a strain-isolated FBG into the same fiber network.

The combined laboratory and field results demonstrate that speckle-based interrogation can function as a reliable, quantitative measurement modality when both the optical element and the computational analysis are expressly engineered for environmental robustness. The stability observed across vibration, acceleration and thermal variation confirms that reproducible speckle generation is achievable in practical settings, not just controlled laboratory conditions. This establishes a viable foundation for deploying engineered speckle interrogation in structural health monitoring scenarios and directly addresses the long-standing criticism that speckle-based spectrometry is inherently too sensitive to external disturbance for real-world use.

## Conclusions

This work demonstrates a fully fiberized, speckle-based spectrometer platform that achieves mechanical and thermal stability not previously reported for such systems. By integrating femtosecond laser-written scattering centers within a high-aspect-ratio flat fiber element and eliminating free-space alignment errors, the STASIS architecture maintains consistent speckle behavior under environmental excitation, including sustained ±7 G accelerations.

Across laboratory and flight testing, the system recovered dynamic strain in the −100 to 400 µε range with standard deviation to measurement of 1.63 µε, and maintained measurement integrity throughout all aircraft maneuvers. The calibrated actuation experiments confirm that PCA-derived strain recovery remains reliable even under vibration-induced perturbations, addressing a long-standing limitation of speckle interrogation, environmentally stability. The system remained operationally stable under a Δ*T* = 35 °C temperature change, exhibiting only deterministic phase drift that was fully corrected in post-processing.

These results establish that engineered speckle interrogation can operate as a practical, compact and low-power alternative to conventional FBG interrogators for dynamic structural health monitoring. The demonstrated stability and fully integrated form factor open a credible pathway to multi-point sensing networks in UAVs, lightweight aerospace structures and other platforms where SWaP-C constraints presently limit conventional photonic instrumentation.

## Methods

### UAV aircraft

The UAV utilized in the tests (Fig. 1a) is a 2-m wingspan bespoke drone, made from LW-PLA (foamed PLA with 60% reduced weight in comparison to standard PLA) wrapped in a fiberglass skin with laser cut plywood formers inside and carbon fiber spars, in the wing and tail. The aircraft loading bay size is 126 *× *70 *× *133 mm. The total power required to take off equals 3.6 kW. The on-board battery with a capacity of 8500 mAh allows approximately 15–20 min of continuous operation with an optional payload of up to 1 kg. The maximum air speed is 42 m/s, and the designed cruise speed is 22 m/s.

### FEA simulation

Finite Element Analysis (FEA) was performed using SolidWorks to evaluate the UAV wing’s structural performance under aerobatic flight conditions. The aircraft was designed in compliance with the EASA CS-23 standard for Aerobatic category aircraft, with a maximum load factor of 9 G, corresponding to 1.5 times the limit load factor of 6.2 G.

Aerodynamic loading was simulated using Ansys Fluent, with the resulting lift distribution (at 9 G) imported into SolidWorks as a distributed load applied to the wing structure. The wing was modeled as a composite laminate (E-glass fiber) with spanwise-varying skin thickness: (1) the wing root of 0.22 mm layer of 100 gsm fiber, (2) the mid-span of 2 layers of 25 gsm fiber (0.1 mm skin thickness), and (3) the wing tip of a single layer of 25 gsm fiber (0.05 mm thickness).

The material properties used were Young’s modulus of 15600 MPa, Poisson’s ratio of 0.36, shear modulus of 8000 MPa, and tensile strength of 280 MPa. The wing root was fixed in the FEA simulation, as this constraint does not affect internal load paths.

The total aerodynamic load of 640 N was applied based on the ultimate load factor of 9 G. The maximum strain did not exceed 2.5%, indicating that the wing maintains structural integrity under the applied loads. The FBG sensors were mounted on the underside of the wing, just outboard of the flaperon, which is the region that experienced the highest strain in the FEA simulations (Fig. 1b).

### Scattering chip fabrication

**Fig. 7:**
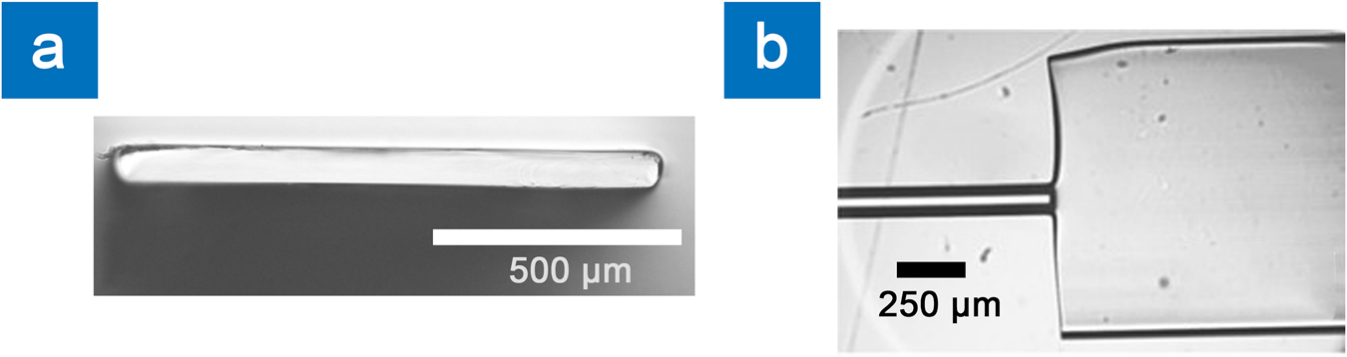
Micrographs of high aspect ratio flat fiber (HARFF)^24^. **a** Cross-section (1000 × 40 µm) and **b** top-down view of fusion splice of regular circular fiber (780HP) to flat fiber^24^.

HARFF was spliced to 780HP fiber using a CO_2_ laser splicer (LZM-100, Fujikura Ltd), producing a monolithic fiber system. In contrast to earlier work^18^, the HARFF used here was fabricated using a capstan-based draw process, enabling continuous fiber lengths of several hundred meters rather than short (~1 m) manually cut sections produced using a tractor-based system. The drawing temperature was increased to approximately 1960 °C, and JGS1 synthetic fused silica was used in place of quartz-derived fused silica. After initial neck-down and once a stable drawing equilibrium was reached, the draw parameters were held constant, with a preform feed rate of 1 mm/min and a capstan speed of 1.5 m/min. These changes resulted in improved geometric consistency and flatness along the fiber length, as evidenced by the cross-sectional profiles shown in Fig. 7a. This geometric uniformity was critical for repeatable splicing and for reducing longitudinal variations in stress sensitivity, which are known to induce speckle instability in scattering-based fiber systems under dynamic excitation.

The CO₂ laser splice was performed by aligning the circular fiber to the HARFF with a nominal 10 µm separation, heating both ends with the CO₂ beam, and then forming the joint by advancing the fibers into contact using a controlled 3 µm hot-push overlap, shown in Fig. 7b.

The scattering array responsible for speckle pattern generation was fabricated within the HARFF substrate using femtosecond laser writing^18^.

### HARFF integration

**Fig. 8:**
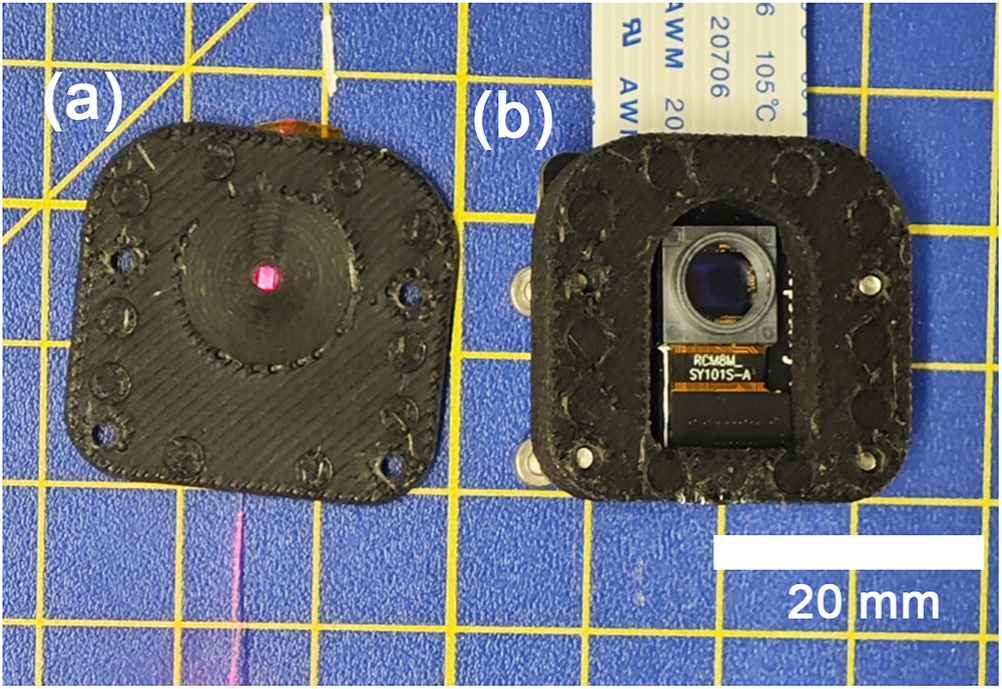
Interrogator sensing head with high aspect ratio flat fiber installed. **a** Base part with imprinted flat fiber and visible scattering array (illuminated with red light); **b** top part with camera sensor (Raspberry Pi V2 NoIR with lens removed).

Following laser inscription, the HARFF was embedded into a sub-assembly component produced using fused deposition modeling 3D printer (Bambu Lab X1C, PLA-CF filament), illustrated in Fig. 8. In contrast to earlier implementations^18^, where the flat fiber was clamped between two printed components, the design presented embeds the HARFF within a single monolithic polymer structure. During printing, molten polymer conforms tightly to the fiber geometry, eliminating discrete mechanical interfaces and suppressing relative motion between the fiber and housing. This monolithic integration is particularly important for scattering-based sensing, where even micron-scale interfacial slip can induce low-frequency speckle drift under vibration. Relative to the earlier clamped configuration, the embedded design exhibited substantially reduced low-frequency speckle drift during vibration excitation, enabling continuous operation without intermittent decorrelation.

The design choice for PLA was primarily driven for reduced mass and ease of fabrication. It should be noted the key optical response is set by the fused-silica fiber and laser-written pattern, which are much stiffer than the surrounding polymer, limiting deformation coupling.

The assembled housing was then incorporated into the STASIS, completing the mechanical integration.

### STASIS specification

The STASIS platform consists of the STASIS head, a broadband light source, Raspberry Pi computer and optical fiber couplers. The whole unit measures 115 *× *63 *× *100 mm and a total weight of 400 g (excluding battery). The device has two FC/APC optical sockets available for connecting optical fibers with FBGs.

The device operation was as follows: fiber-coupled input light from a superluminescent diode (SLED) with 810 nm central wavelength and 30 nm 3 dB bandwidth (SLD810S, Thorlabs) mounted on SF8025-ZIF14 controller board (Maiman Electronics) was coupled into one port of a 2 × 2 50:50 splitter. Ports 2 and 3 on the other side of the coupler were used as the two connection sockets for each FBG. The HARFF with a scattering matrix was connected to the fourth port. Thereby, the counterpropagating Bragg wavelength(s) upon returning through the splitter is launched into the flat fiber scattering matrix. In the latter, the reflected spectrum is converted to speckle patterns and recorded by a Raspberry Pi V2 NoIR camera with lenses removed (Sony IMX219 CMOS diagonal sensor (4.60 mm diagonal), unit cell size: 1.12 × 1.12 µm, number of active pixels: 3280 × 2464). The images were captured in native-binning mode, with output resolution of 640 × 480 (approx. 5× pixel binning). The exposure parameters were fixed at 0.1 s shutter speed and sensor ISO = 100. The system operation and on-board computation was carried out an on-board Raspberry Pi Compute Module 4. Lastly, interrogation results were stored locally and also remotely transmitted via MAVLink protocol to the autopilot computer of the UAV. The results were then further streamed to the ground station, thus providing live tracking of the FBG readings.

For the flight tests, STASIS unit was installed inside the payload bay of the fuselage securing with Velcro straps. The fibers with FBGs were routed into the fuselage through access points and connected to the unit via FC/APC connectors. The system was powered by an auxiliary battery (5 V, 1.5 A). After closing the payload bay hatch and securing it, the drone was left for a 30 min system warm up and thermal equilibration.

### FBG characterization and mounting

**Fig. 9:**
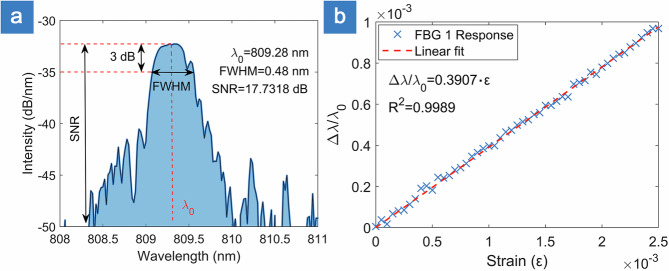
Fiber Bragg grating (FBG) characterization example for FBG 1. **a** Reflected power spectrum analysis and **b** strain-induced wavelength shift response. FWHM Full-width at half-maximum linewidth, SNR signal-to-noise ratio.

For the UAV test flights, two FBGs (each 5 mm long) were laser-written in separate sections of 780HP fiber (each grating was inscribed in its own individual fiber). Their reflection spectra were characterized using an optical spectrum analyzer (OSA Yokogawa AQ6370D), and the strain response was evaluated by mounting 5 cm fiber sections on linear translation stages (MAX381, Thorlabs) and stretching them longitudinally from 0 to 2500 µm in 50 µm increments (Fig. 9). The first FBG exhibited a Bragg wavelength of 809.28 nm, a 0.48 nm FWHM, and an SNR of 17.7 dB, yielding a strain-sensitivity coefficient *k* of 0.3907. The second FBG showed a Bragg wavelength of 809.62 nm, a 0.34 nm FWHM, and an SNR of 17.4 dB, with a corresponding *k* value of 0.3905.

After the initial characterization, the FBGs were attached to the underside of the aircraft wing, near flaperons, using epoxy adhesive (LOCTITE EA3430). The specific location was determined through finite element analysis, to identify the area where the structure would experience the greatest strain during the flight (Fig. 1b).

### Speckle pattern dissimilarity

The applied strain experienced by the FBG can be reconstructed by a number of statistical techniques. This paper focuses on image dissimilarity and principal component analysis (described in section below). These two metrics are also used to evaluate the stability of the system during the vibration tests.

Changes in the speckle pattern intensity profiles are evaluated by defining the similarity *S* as the absolute value of the dot product between average-subtracted (subtracting arithmetic mean of 300 samples of idle state baseline DC-level) and normalized (Euclidean norm) vectors (a, b) of reference and investigated images (Eq. 2). This similarity always yields a result between 0 and 1.

2 $$S=\left|\frac{a-{{{\rm{DC}}}}_{{{\rm{mean}}}}}{\left|\left|a-{{{\rm{DC}}}}_{{{\rm{mean}}}}\right|\right|}\cdot \frac{b-{{{\rm{DC}}}}_{{{\rm{mean}}}}}{\left|\left|b-{{{\rm{DC}}}}_{{{\rm{mean}}}}\right|\right|}\right|$$

If the result is 0, it means the vectors (images) are perpendicular and there is no similarity between them. On the contrary, if similarity equals 1, the vectors are parallel (identical). For the case of speckle patterns here, which show minor changes in intensity distribution for the range of strain values investigated, it is expected that similarity will be close to its upper limit^20^.

Therefore, since the speckle patterns investigated in this paper are very similar to each other, the new quantity is introduced which represents image dissimilarity scaled up by 1000, i.e., (1−S) × 1000. In this manner numerical results obtained are intuitive and easy to follow if image similarity/dissimilarity changes.

In this paper dissimilarity is calculated using the initial frame or baseline as a reference, hence the metric is referred as Baseline Frame Dissimilarity (BFD).

### Principal component analysis (PCA)

The PCA reconstruction process requires calibration, a collection of previously registered speckle patterns, corresponding with known and defined state of system input. By appending each vectorized speckle pattern next to the previous one, a 2D calibration matrix can be formed, where each column contains information about one given system input (sensed quantity)^21,22^. The cornerstone of this method is extracting principal components (PC)—i.e., directions in which the newly projected data variance will be the highest, thus conveying the most useful information^23^. This operation is performed on calibration matrix results in PC matrix (*V*), where its rows correspond to the number of dimensions in the system (i.e., total pixel number) and columns to the number of available principal components (i.e., data projection directions). By multiplying input column vector(s) of speckle(s) (*P*) with principal components, the data will be projected—i.e., represented in a low-dimensionality, high-variance space (Eq. 3).

3 $${P}_{{{\rm{proj}}}}={P}^{T}\cdot V$$

where *T* is a transpose operator (switching rows-columns of matrix) and *P*_proj_ is the projected data matrix. The results can be visualized by selecting column of *P*_proj_ (given principal component projection), since the number of rows will correspond to the number of observed speckles. Therefore, the number of data dimensions can be successfully reduced from the total number of pixels of speckle pattern image to just 1D point.

In reconstructive spectrometry, PCA is often used to eliminate the effect of external factors as multiple variables can be effectively distributed across different PCA components providing noise free signal. This approach, however, requires construction of reliable calibration set or pass whole measured data through decomposition algorithm, which is very computationally intense process.

Finally, by recalling that the PCA-projected data is unitless (i.e., projection reveals data trend in unitless, abstract space), to recover the measurand (strain) the projection values of PCA (in most cases first Principle Component, PC1, since it is the most contributing component) must be scaled with the proportionality coefficient which links the a-priori known measurand values with the PC projections, hence converting the unitless, measurands to well-understood unit space.

### Thermal and vibration test setup and protocol

**Fig. 10:**
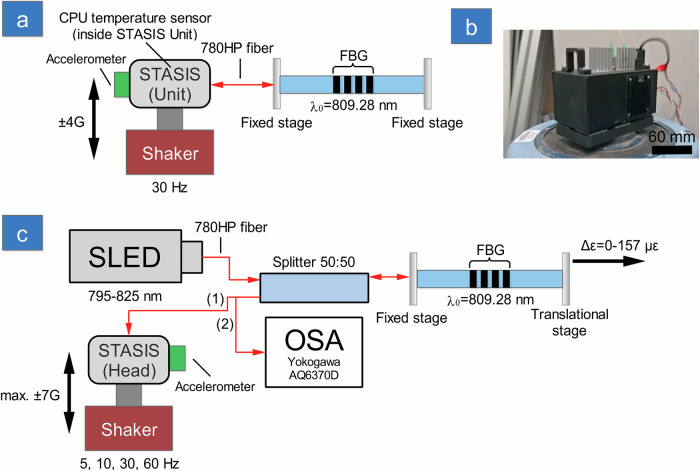
Experimental test set ups for evaluating performance of speckle-based tracking and stabilized interrogation system (STASIS). **a** Diagram of STASIS thermal test; **b** photo of STASIS unit on shaker and **c** schematic of STASIS head vibration test. Shaker waveform generator and power amplifier are not shown for clarity. SLED superluminescent diode, OSA optical spectrum analyzer, FBG fiber Bragg grating, CPU central processing unit.

Thermal test evaluates the stability of speckle images while the FBG is under constant tension and the STASIS unit (integrated interrogator) is subjected to vibrations, lasting approximately 50 min. During testing, BFD values were recorded against time lapsed, together with Raspberry Pi CPU temperature and vertical acceleration (schematically presented in the Fig. 10a).

The STASIS unit was mounted on the top of the mechanical shaker and secured with mounting screws and clamps to prevent vibration-driven displacement (Fig. 10b).

The test was divided into three stages: (1) system boot-up and warm-up (30 min); (2) mechanical agitation at 30 Hz frequency and *±*4 G amplitude acceleration (20 mins); (3) system shut-down. During the agitation step, the electronic accelerometer (The Cube Orange) sampled the acceleration at 50 Hz with a “baseline” of −1 G (negative represents the acceleration towards the ground and positive in the opposite direction).

After thermal test was terminated, the CPU temperature and BFD data were analyzed, and the temperature model used for detrending was identified (Fig. 4).

To assess the stability, the BFD and PC1 metrics were calculated using speckle images captured during vibration tests using a shaker setup, with an FBG which is either static or under varying applied strain. The STASIS platform sensing head was mounted on the top of a mechanical shaker (VP4, Derritron) and secured with mounting screws to prevent displacement due to vibrations. On the side of STASIS head, the external accelerometer (LIS3DH, Adafruit) was attached to monitor vibration conditions. During the vibration test, this allowed the vertical acceleration to be recorded at a sampling frequency of 360 Hz.

The shaker is driven by sinusoidal wave at frequencies ranging from 5 to 60 Hz driven by a waveform generator (TG210, Thurlby) after passing through 3 W power amplifier (SignalForce, DataPhysics). The amplification level was set to produce maximum *±*7 G of acceleration registered by external accelerometer. The schematic of the experimental set-up is presented in the Fig. 10c.

The input light is generated by SLED (810S, Thorlabs) and sent to the 50:50 Splitter (BXC53, Thorlabs). After passing the splitter, the light reaches the FBG, where the back reflected light returns through the splitter to the STASIS Head (connection 1, Fig. 10c—speckle pattern-based experiments) or OSA (connection 2, Fig. 10c—obtaining Bragg wavelength peak shift during stage tension movement with no shaking (OSA-based calibration)).

During the shaker tests, an FBG was suspended by mounting both ends of the fiber on a computer-controlled linear stage (MAX381, Thorlabs). The vibration experiment consisted of four cycles, when the shaker was operated at either 5, 10, 30, or 60 Hz. Each cycle included three sub-cycles, with only the middle sub-cycle involved shaking of the STASIS. In every sub-cycle, the tensile sequence was repeated five times, applying tension from 0 to 157 μɛ and then releasing back to 0 μɛ. For each tension and release step, 20 speckle patterns were recorded, giving 60 images per repetition. After completing all sub-cycles, the entire procedure was repeated for all shaker vibration frequencies. Speckle patterns were captured at 10 Hz and subsequently analyzed using BFD and PCA algorithms.

## Data Availability

The datasets generated during this study are openly available in the University of Southampton repository (10.5258/SOTON/D3800).
